# Development and evaluation of a Pan-Mucorales Real-time PCR and a multiplex Real-time PCR for detection and identification of *Rhizopus arrhizus, Rhizopus microsporus*, and *Mucor* spp. in clinical specimens

**DOI:** 10.1128/jcm.01937-24

**Published:** 2025-04-30

**Authors:** Helen M. Tang, Sharon C.-A. Chen, Kerri Basile, Catriona L. Halliday

**Affiliations:** 1 Centre for Infectious Diseases and Microbiology Laboratory Services, Institute of Clinical Pathology and Medical Research, NSW Health Pathology, Westmead Hospital 631947, Westmead, New South Wales, Australia; 2 Faculty of Medicine and Health, The University of Sydney, Camperdown, New South Wales, Australia; 3 Sydney Infectious Diseases Institute, The University of Sydney, Westmead, New South Wales, Australia; University of Calgary, Calgary, Alberta, USA

**Keywords:** Mucorales, PCR, *Rhizopus arrhizus*, *Rhizopus microsporus*, *Mucor* spp., *Mucormycosis*

## Abstract

Mucormycosis is a life-threatening infection associated with high morbidity and mortality. Rapid and accurate diagnosis is essential for improving patient outcomes. Conventional diagnostic methods, such as histopathology and culture, are limited by low sensitivity and prolonged turnaround times, while commercial polymerase chain reaction (PCR) assays are costly and may lack specific genus or species targets. Here, we present a novel molecular diagnostic workflow to facilitate the rapid detection of Mucorales directly from clinical specimens. This workflow integrates two in-house *in vitro* diagnostic PCR assays: a real-time, qualitative Pan-Mucorales PCR, followed by a real-time multiplex genus/species-specific PCR targeting *Rhizopus arrhizus*, *Rhizopus microsporus*, and *Mucor* spp. Specificity of the assays was validated using cultured isolates of Mucorales, as well as non-Mucorales fungi and bacteria. The diagnostic performance was assessed across 166 clinical specimens (70 Mucorales-positive and 96 negative), confirmed by an in-house panfungal PCR and DNA sequencing protocol. Specimens studied included fresh and formalin-fixed paraffin-embedded tissues, fluid, bronchoalveolar lavage/washing fluid, fine needle aspirate, cerebrospinal fluid, and bone. The Pan-Mucorales PCR demonstrated 98.6% sensitivity and 100% specificity, while the multiplex genus/species-specific PCR assay yielded sensitivities of 93.8% for *R. arrhizus*, 70.8% for *R. microsporus*, and 75% for *Mucor* spp., each with 100% specificity. Concordance with the panfungal PCR (>99% for Pan-Mucorales PCR and >89% for multiplex PCR) was high, supporting the robustness of the workflow. This diagnostic approach has the potential to significantly reduce turnaround times, labor and costs, while streamlining the diagnostic process through timely, precise diagnostics.

**IMPORTANCE:**

Mucorales fungi, identified collectively as a high-priority pathogen on the World Health Organization fungal priority pathogens list, are the causative agents of mucormycosis. Mortality is high (up to 80%), and early, accurate diagnosis is critical to enable timely initiation of targeted antifungal therapy and surgical debridement for source control to optimize patient outcomes. In our laboratory, as in many others, the current standard for the diagnosis of mucormycosis is histopathology and culture-based methods supplemented by panfungal PCR assay/DNA sequencing; however, this process may take 7 days, with considerable labor and cost implications. Here, we present two Mucorales-specific real-time PCR assays, which when used sequentially, reduce diagnostic turnaround time and costs to detect three common agents of mucormycosis—*Rhizopus microsporus*, *Rhizopus arrhizus*, and *Mucor* species. This approach not only improves diagnostic efficiency and integration into workflow but can facilitate surveillance through accurate genus- and species-level identification.

## INTRODUCTION

The Mucorales pose a significant threat to humans due to their ability to cause rapidly progressive angioinvasive infections associated with high mortality (up to 80%) (1). Worldwide, the predominant Mucorales associated with mucormycosis include *Rhizopus* species (spp.), *Mucor* spp., *Lichtheimia* spp., and *Rhizomucor* spp., with *Cunninghamella* spp., *Apophysomyces* spp., and *Saksenaea* spp. less common, although their prevalence varies by geographic region (1). In a multicenter Australian study of mucormycosis, *Rhizopus* (including *Rhizopus arrhizus* and *Rhizopus microsporus*) and *Mucor* spp. were the leading causative genera, with a 180-day mortality rate of 56.7% (2). Risk factors for invasive mucormycosis are well known and include uncontrolled diabetes mellitus, haematological malignancy, solid organ transplantation, corticosteroid use, major trauma in otherwise immunocompetent individuals, and most recently, preceding coronavirus disease 2019 infection (3–6). The incidence of mucormycosis appears to be rising, driven in part by aggressive immunosuppressive therapies and expanded use of triazole antifungal prophylaxis among haematological patients (7) and increasing prevalence of poorly controlled diabetes (8).

The gold standard for diagnosis of mucormycosis relies on identifying characteristic ribbon-like, sparsely septate, irregularly branching hyphae in histopathological tissue sections with confirmation on culture from a normally sterile site (9). However, accurate histopathological diagnosis relies on expertise and does not allow for pathogen speciation (10). Culture-based methods face obstacles due to hyphal fragility and reduced sensitivity in patients receiving antifungal therapy. Hence, the diagnosis of mucormycosis is often delayed or missed, with a significant proportion of cases identified post-mortem (11). The absence of approved Mucorales-specific serological tests further limits diagnosis (12, 13).

Recent advancements in molecular techniques have significantly enhanced the diagnosis of mucormycosis, with the introduction of commercial assays such as MucorGenius (PathoNostics, Netherlands), Fungiplex *Mucorales* RUO Real-Time PCR Kit (Bruker, Bremen, Germany), and MycoGENIE *Aspergillus-Mucorales* spp. (Ademtech, Pessac, France). However, these assays are costly and only detect the Mucorales order without providing species-level identification (14, 15). Our laboratory currently relies on culture-based approaches supplemented by panfungal PCR/DNA sequencing of tissue and other specimens such as bronchoalveolar lavage fluid (BALF) specimens from high-risk patients; the latter takes up to 7 days for a result.

To expedite the diagnosis of mucormycosis, here we aimed to develop and evaluate two sequential real-time PCR assays—(i) a Pan-Mucorales PCR to enable rapid detection of Mucorales fungi, and (ii) a multiplex genus/species-specific PCR assay to specifically identify *R. arrhizus, R. microsporus,* and *Mucor* spp.—in clinical specimens. The genus/species-specific targets were chosen based on what is known about the epidemiology of mucormycosis in Australia (2) and for best guess capture of the potential causative pathogen. The two assays were further designed to enable a streamlined diagnostic workflow, with the goal of reducing test turnaround time (TAT), staff labor, and costs.

## MATERIALS AND METHODS

### Fungal isolates and bacteria

A total of 80 fungal and 10 bacterial isolates were studied to evaluate the sensitivity and specificity of both Mucorales PCR assays (Supplementary Materials, Appendices 1 and 2). These comprised R. *arrhizus* (*n* = 11), *R. microsporus* (*n* = 12), and *Mucor* spp. (*n* = 11), as well as a variety of other Mucorales (*n* = 22) and non-Mucorales fungal pathogens (*n* = 24) (Table 1). Specificity testing for non-culturable *Pneumocystis jirovecii* was conducted using a clinical specimen known to contain *P. jirovecii* DNA (i.e., PCR-positive). The identification of all fungal isolates was confirmed by sequencing of the internal transcribed region (ITS) region of rDNA (16). The bacterial pathogens (*n* = 10) studied are shown in Table 1 and were identified by matrix-assisted laser desorption ionization-time of flight mass spectrometry (MALDI-TOF MS, Bruker Daltonics, Bremen, Germany). All “test” pathogens were chosen due to their frequent presence in clinical specimens relevant to the clinical context where the assays may be requested. All bacterial isolates apart from *Streptococcus oralis/mitis, Streptococcus dysgalactiae,* and *Moraxella catarrhalis* were American Type Culture Collection control strains.

**TABLE 1:** Fungi and bacteria studied

Mucorales fungi (*n* = 56)	Non-Mucorales fungi (*n* = 24)	Bacterial isolates (*n* = 10)
*Cunninghamella bertholletiae* (*n* = 1)	*Alternaria* spp. (*n* = 1)	*Staphylococcus aureus* (*n* = 1)
*Cunninghamella* spp*.* (*n* = 1)	*Aspergillus calidoustus* (*n* = 1)	*Haemophilus influenzae* (*n* = 1)
*Lichtheimia corymbifera* (*n* = 6)	*Aspergillus flavus* complex (*n* = 1)	*Streptococcus pyogenes* (*n* = 1)
*Lichtheimia ramosa* (*n* = 5)	*Aspergillus fumigatus* sensu stricto (*n* = 1)	*Streptococcus pneumoniae* (*n* = 1)
*Mucor amphibiorum* (*n* = 1)	*Aspergillus nidulans* complex (*n* = 1)	*Streptococcus oralis/mitis* (*n* = 1)
*Mucor circinelloides* (*n* = 7)	*Aspergillus niger* complex (*n* = 1)	*Streptococcus dysgalactiae* (*n* = 1)
*Mucor irregularis* (*n* = 1)	*Candida albicans* (*n* = 1)	*Escherichia coli* (*n* = 1)
*Mucor ramosissimus* (*n* = 1)	*Cladophialophora bantiana*	*Pseudomonas aeruginosa* (*n* = 1)
*Mucor* spp*.* (*n* = 1)	*Conidiobolus* spp. (*n* = 1)	*Klebsiella pneumoniae* (*n* = 1)
*Rhizomucor miehei* (*n* = 1)	*Cryptococcus gattii* (*n* = 1)	*Moraxella catarrhalis* (*n* = 1)
*Rhizomucor pusillus* (*n* = 1)	*Cryptococcus neoformans* var. *grubii* (*n* = 1)	
*Rhizopus arrhizus* (*n* = 11)	*Exophiala dermatitidis* (*n* = 1)	
*Rhizopus microsporus* (*n* = 12)	*Exophiala oligosperma* (*n* = 1)	
*Saksenaea vasiformis* complex (*n* = 2)	*Fusarium oxysporum* complex (*n* = 1)	
*Syncephalastrum racemosum* (*n* = 5)	*Fusarium solani* complex (*n* = 1)	
	*Lomentospora prolificans* (*n* = 1)	
	*Nakaseomyces glabratus* (*n* = 1)	
	*Penicillium* spp. (*n* = 1)	
	*Pneumocystis jirovecii* (*n* = 1)^*a*^	
	*Scedosporium apiospermum* (*n* = 2)	
	*Scedosporium aurantiacum* (*n* = 1)	
	*Scedosporium boydii* (*n* = 1)	
	*Talaromyces* spp. (*n* = 1)	

^*a*^ Tested using clinical specimen.

### Clinical specimens

**Fig 1:**
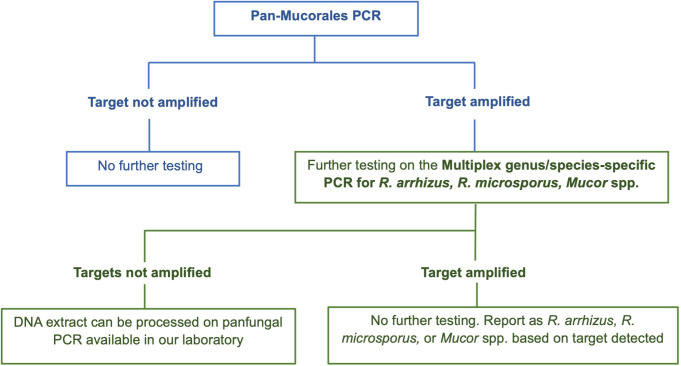
Testing algorithm for Pan-Mucorales and multiplex PCRs.

Archived clinical specimens (*n* = 166) tested for fungi as part of routine standard of care collected between November 2013 and September 2024 were evaluated by the two PCR assays (Supplementary Material, Appendix 3). Various specimen types, either known to contain (*n* = 70) or not contain Mucorales DNA (*n* = 96) based on previous panfungal PCR testing, were studied. These included fresh tissue (*n* = 72), formalin-fixed paraffin-embedded (FFPE) tissue (*n* = 23), fluid (*n* = 29), BALF/bronchial washing (BW) (*n* = 28), cerebrospinal fluid (CSF) (*n* = 6), bone (*n* = 3), fine needle aspirates (FNA) (*n* = 2), bone marrow (*n* = 1), sputum (*n* = 1), and unspecified (*n* = 1). Specimens were stored under various conditions (4°C, −20°C, and −70°C) as part of routine laboratory practice. The PCR results from the two newly developed assays were compared with results of fungal culture, histopathology, panfungal PCR, and DNA sequencing where available. The Mucorales-specific PCR assays were performed following the testing algorithm shown in Fig. 1.

### DNA extraction

DNA from fungal cultures was extracted using NucliSens EasyMag (bioMérieux Inc., Durham, North Carolina, USA) following the manufacturer’s instructions. DNA was extracted from bacterial isolates using a boiling technique whereby two bacterial colonies were suspended in 1 mL of sterile water, boiled for 10 min at 100°C, and subsequently centrifuged for 5 min at 1,000 rpm (17); 10 µL of the supernatant was used for each PCR reaction.

DNA extraction from clinical samples was performed using the High Pure PCR Template Preparation Kit (Roche Diagnostics, Mannheim, Germany). An in-house manual extraction method using histolene was performed to remove paraffin from FFPE tissue prior to DNA extraction, as described by Lau et al. (16). DNA extracts were stored at either −20°C or −70°C prior to PCR testing.

### Primers and probes

Primer and probe sequences targeting the 18S rDNA gene (Table 2), as described by Springer et al*.* (12), were selected for the Pan-Mucorales PCR assay . For the multiplex genus/species-specific assay, primers and probes described by Bernal-Martinez et al. (18) targeting the ITS1 region were chosen for *R. arrhizus* and *R. microsporus* (Table 2). *Mucor*-specific primer and probe sequences targeting the ITS region were selected from sequences published by Lengerova et al*.* (19) (Table 2). All primer and probe combinations were assessed for specificity using the National Centre for Biotechnology Information Primer Basic Local Alignment Search Tool (BLAST) (https://blast.ncbi.nlm.nih.gov/Blast.cgi).

**TABLE 2:** Primer and probe sequences^*a*^

	Sequence (5′–3′)
**Pan-Mucorales PCR**	
PanMuc18 F	TTACC**R**TGAGCAAATCAGA**R**TG
PanMuc18 R	AATC**Y**AAGAATTTCACCTCTAGCG
PanMuc18 P	HEX-T**YRR**(G)G(G)**B**(A)T(T)T(G)T(A)TTT-BHQ1
**Multiplex genus/species-specific PCR**	
Rharr2 F	TCTGGGGTAAGTGATTGC
Rharr2 R	GCGAGAACCAAGAGATCC
Rharr2 P	Cy5-CGCGATAACCAGGAGTGGCATCGATCAAATCGCG-BHQ2
Rhmic2 F	CTTCTCAGTATTGTTTGC
Rhmic2 R	ATGGTATATGGTAAAGGG
Rhmic2 P	HEX-CGCGATCCTCTGGCGATGAAGGTCGTATCGCG-BHQ1
Muc F	GCAACTTGCGCTCATTGGTA
Muc R	GGATAGAGGGTTTGTTTTGATACTGAA
Muc P	6-FAM-CCAATGAGCACGCCTG-BHQ1
**HBG PCR** ^***b***^	
HBG F	GAAGAGCCAAGGACAGGTAC
HBG R	CACCAACTTCATCCACGTTCAC
HBG P	TxRd-TCAAACAGACACCATGGTGCACCTG-BHQ2

^*a*^ **R** stands for A or G, **Y** stands for C or T, and **W** stands for A or T; F, forward primer; HBG, human β globin; Muc, *Mucor* spp.; P, probe; PCR, polymerase chain reaction; R, reverse primer; Rharr, *Rhizopus arrhizus*; Rhmic, *Rhizopus microsporus*.

^*b*^ HBG PCR was incorporated into both the Pan-Mucorales and multiplex genus/species-specific PCR assays.

Probes within the assays were labeled with distinct fluorescent dyes, allowing for accurate detection in separate channels of the LightCycler 480 II instrument (Roche Diagnostics). Both assays were multiplexed with a human β-globin (HBG) primer and probe, serving as an internal control to detect PCR inhibition and interference and verification of specimen adequacy. Each primer and probe set was first evaluated in a singleplex format before being incorporated into the multiplex assay with HBG.

### Real-time PCR

PCRs were performed in a final volume of 25 µL. The Pan-Mucorales PCR contained 1× SensiFAST Probe No-ROX Master Mix (Bioline, Meridian Bioscience, London, United Kingdom), 0.5 µM PanMuc18 forward and reverse primers, 0.2 µM PanMuc18 probe, 0.5 µM of the HBG forward and reverse primers, 0.2 µM HBG probe, and 10 µL DNA. The Mucorales multiplex genus/species-specific PCR contained 1× SensiFAST Probe No-ROX Master Mix, 0.5 µM each of *R. arrhizus*, *R. microsporus*, *Mucor* spp., and HBG forward and reverse primers, 0.2 µM each of *R. arrhizus*, *R. microsporus*, *Mucor* spp. probes, 0.1 µM HBG probe, and 10 µL DNA.

PCR amplification was carried out on the LightCycler 480 II instrument (Roche Diagnostics) as follows: an initial denaturation at 95°C for 10 min, followed by 50 cycles of 95°C for 15 s and 60°C for 60 s with single acquisitions made after each 60°C stage, and a final cooling step at 40°C for 30 s. A positive result was defined as a crossing point (C_p_) value of <40 cycles after applying color compensation analysis. All PCR curves were manually reviewed to confirm that the curve shape and end-point fluorescence were consistent with a true positive result.

DNA extracts from spiked BALF containing 10^5^ CFU/mL and 10^3^ CFU/mL culture suspensions of *R. arrhizus, R. microsporus*, and *Mucor* spp. were used as high and low positive controls, respectively. They were included in each PCR run to ensure assay sensitivity and for the detection of amplification inhibition. Sterile nuclease-free water served as a no-template control in each run to monitor for contamination.

### Limit of detection

Suspensions of *R. arrhizus, R. microsporus, Mucor* spp., and *Lichtheimia corymbifera* conidia were prepared in sterile water and adjusted to an absorbance of 0.15–0.17 at 530 nm, which corresponds to 0.2–2.5 × 10^6^ CFU/mL. To determine the limit of detection, serial dilutions ranging from 10^4^ to 10^1^ CFU/mL were prepared and spiked into BALF, and DNA was extracted as outlined above (Supplementary Material, Appendix 4). Prior to spiking, these BALFs were pooled and confirmed to be free of Mucorales DNA through panfungal PCR testing. The concentration of the inoculum was confirmed by plating 10 µL of the 10^3^ and 10^2^ dilutions on Sabouraud’s dextrose agar. The limit of detection was defined as the lowest BALF concentration in which the target was detected by PCR.

### Analysis

Sensitivity, specificity, analytical positive predictive value (PPV), and negative predictive value (NPV) for each PCR assay were assessed against the panfungal PCR, the current molecular standard for Mucorales diagnosis. Notably, the PPV and NPV calculated here reflect the analytical performance of the assays rather than clinical predictive values, which require dedicated clinical studies. Additionally, the overall percentage agreement was calculated relative to the panfungal PCR results.

## RESULTS

### Fungal isolates and bacteria

The Pan-Mucorales PCR assay demonstrated 100% sensitivity and specificity when tested on 56 Mucorales fungal isolates, 24 non-Mucorales fungi, and 10 bacterial pathogens (Supplementary Material, Appendix 1).

The Mucorales multiplex genus/species-specific PCR assay displayed 100% sensitivity for both *R. arrhizus* and *R. microsporus*, detecting all 11/11 and 12/12 culture isolates, respectively (Supplementary Material, Appendix 2). No interspecies cross-reactivity was observed for either *Rhizopus* target. Sensitivity for the *Mucor* spp. was lower at 91% (10/11), with the PCR not able to detect and identify *Mucor amphibiorum*. Specificity in all cases was 100%, with no cross-reactivity observed between species or genera for cultured isolates or for non-targeted Mucorales species or other fungi, and in particular, no amplification with any of the bacteria tested.

### Limit of detection

The limit of detection of the Pan-Mucorales PCR assay was approximately 10^1^ CFU/mL, determined using BALF spiked with conidial suspensions of *R. arrhizus, R. microsporus, Mucor* spp., and *L. corymbifera* at final concentrations ranging from 10^3^ to 10^0^ CFU/mL (Supplementary Material, Appendix 4). When assessed on the targeted PCR assay, the limit of detection of all three targets (*R. arrhizus, R. microsporus, Mucor* spp.) was also 10^1^ CFU/mL (Supplementary Material, Appendix 4).

### Clinical specimens

**Fig 2:**
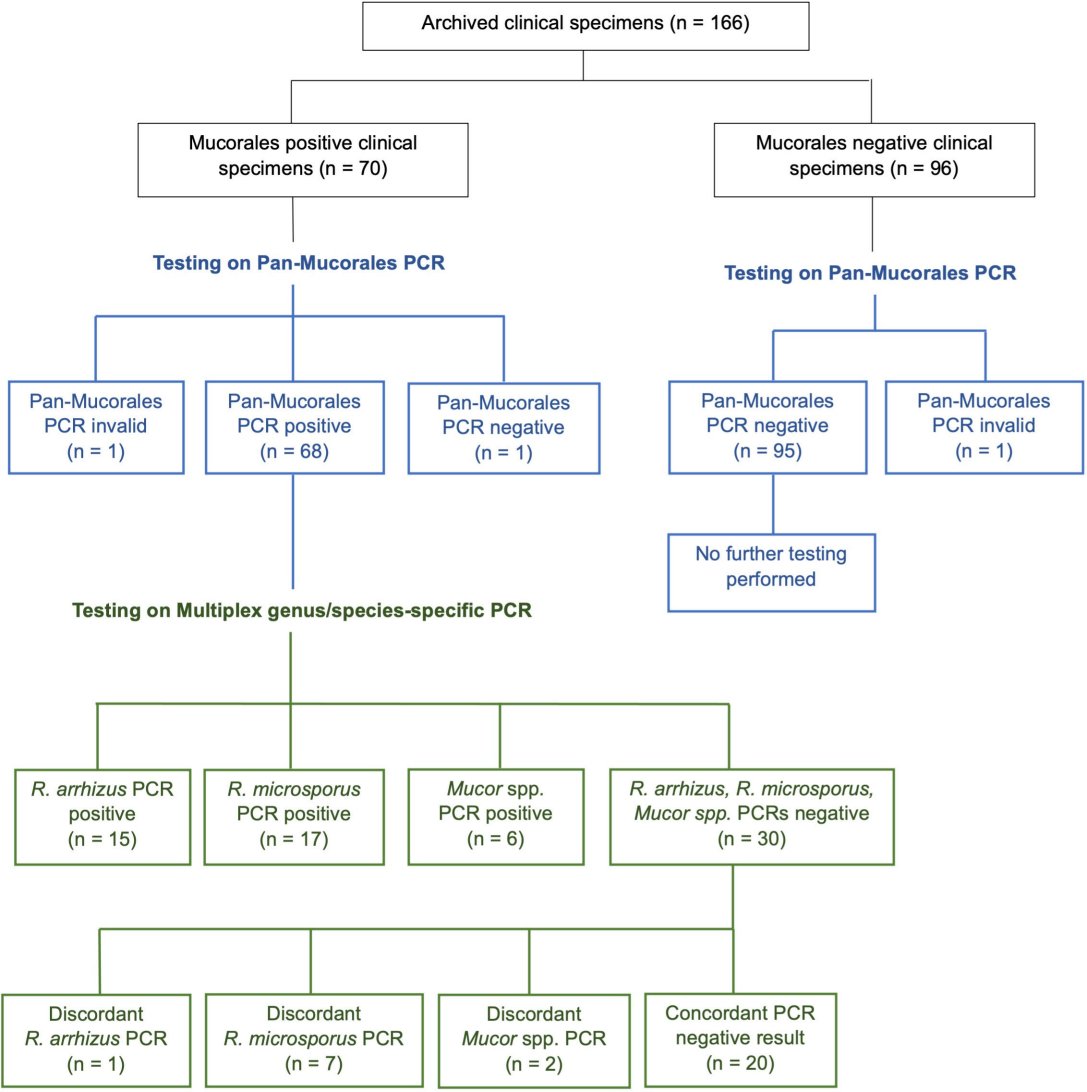
Results of testing archived clinical specimens.

Out of the 166 clinical specimens evaluated (Supplementary Material, Appendix 3), as shown in Fig. 2, 164 were included in the final analysis. Two FFPE tissue samples were excluded due to invalid results, likely caused by sample inhibition or DNA degradation.

The Pan-Mucorales PCR assay demonstrated 98.6% sensitivity (68/69) and 100% specificity (95/95) (Table 3). The single discordant (false negative) result occurred in a sputum sample collected in 2021, likely due to Mucorales DNA degradation as a result of prolonged storage at −70°C. The median C_p_ value was 27.20 (range 8.3–36.22) for positive results, with a median C_p_ of 25.86 (range 17.92–36.70) for HBG. Analytical positive and negative predictive values were calculated to be 100% and 99%, respectively (Table 3). Furthermore, results from the Pan-Mucorales PCR assay demonstrated 99.4% agreement with those obtained by panfungal PCR.

**TABLE 3:** Evaluation of clinical samples using the Pan-Mucorales and Mucorales multiplex genus/species-specific PCR assays

	Pan-Mucorales PCR	Multiplex genus/species-specific PCR		
		*R. arrhizus* PCR	*R. microsporus* PCR	*Mucor* spp. PCR
Sensitivity	98.6% (68/69)	93.8% (15/16)	70.8% (17/24)	75% (6/8)
Specificity	100% (95/95)	100% (52/52)	100% (44/44)	100% (60/60)
Positive predictive value	100% (68/68)	100% (15/15)	100% (17/17)	100% (6/6)
Negative predictive value	99.0% (95/96)	98.1% (52/53)	86.3% (44/51)	96.8% (60/62)
Percentage agreement with panfungal PCR	99.4% (163/164)	98.5% (67/68)	89.7% (61/68)	97.1% (66/68)

Following the testing algorithm (Fig. 1), 68 specimens with Mucorales DNA were further analyzed using the multiplex genus/species-specific PCR assay (Supplementary Material, Appendix 3). Clinical sensitivity of the *R. arrhizus* PCR target was 93.8% (15/16), with one false negative result from a fresh tissue sample (Table 3). The *R. microsporus* PCR target demonstrated a lower sensitivity of 70.8% (17/24), with seven discordant results, mainly from fresh tissue and BALF. The *Mucor* spp. PCR target demonstrated 75% (6/8) sensitivity, with two discordant results from a fresh tissue and a FFPE tissue specimen, both containing *M. amphibiorum.* All specimens containing human pathogenic *Mucor* spp. were detected. The median C_p_ value was 27.40 (range 21.04–36.55) for positive *R. arrhizus* results, 28.19 (range 12.34–36.69) for positive *R. microsporus* results, and 31.30 (range 13.6–34.15) for positive *Mucor* spp. results. The median C_p_ for HBG on the multiplex PCR was 27.41 (range 19.18–38.51). Clinical specificity for the Mucorales multiplex assay was 100% for all three targets. The analytical PPV was calculated to be 100% for all targets, with NPVs ranging from 86.3% to 98.1%. The overall agreement with panfungal PCR was 98.5% for *R. arrhizus*, 89.7% for *R. microsporus*, and 97.1% for *Mucor* spp. (Table 3). Twenty of the 68 specimens evaluated on the multiplex assay contained Mucorales DNA from species other than *R. arrhizus, R. microsporus*, and *Mucor* spp. None of these were detected on the multiplex assay, as expected. The sequencing of Pan-Mucorales PCR amplicons, which were only 90–100 bp in length, matched species identifications made by the panfungal PCR.

For clinical specimens that tested positive by either or both PCR assays, results were compared with other diagnostic methods (Table 4). However, corresponding culture and histology data were unavailable for most samples. Positive results are expressed as a percentage of the total number of samples tested (*n* = 70) in Table 4. Overall, the Pan-Mucorales PCR detected all samples that were positive by culture or histology (*n* = 24). After excluding four samples containing Mucorales fungi not targeted by the multiplex PCR, the targeted assay detected 85% (17/20) of the remaining samples. The three discrepant results were from fresh tissue specimens that contained low levels of *R. microsporus* DNA, as indicated by high C_p_ values (>30) on the Pan-Mucorales PCR. Overall, the findings indicate that the Pan-Mucorales PCR effectively detects and confirms the presence of Mucorales DNA in histology- and culture-positive specimens, while the targeted multiplex assay appears to be less sensitive. Further large-scale studies comparing Pan-Mucorales and targeted PCR assays against culture and histology across diverse specimen types are needed to establish the relative sensitivity of the PCR assays in detecting Mucorales compared to traditional methods.

**TABLE 4:** Pan-Mucorales and multiplex genus/species-specific PCR compared with other diagnostic modalities^*a*^

Specimen type	Culture positive	Histology	Panfungal PCR	Pan-Mucorales PCR	Targeted multiplex genus/species-specific PCR
Fresh tissue (*n*= 38)	4 (11%)	11 (30%)	38 (100%)	38 (100%)	23 (61%)
FFPE tissue (*n* = 14)	NA	7 (50%)	14 (100%)	13 (93%)	8 (57%)
BALF/BW (*n* = 6)	NA	NA	6 (100%)	6 (100%)	2 (33%)
Fluid (*n* = 6)	1 (17%)	NA	6 (100%)	6 (100%)	2 (33%)
FNA (*n* = 1)	NA	NA	1 (100%)	1 (100%)	0 (0%)
CSF (*n* = 2)	NA	NA	2 (100%)	2 (100%)	2 (100%)
Bone (*n* = 1)	NA	1 (100%)	1 (100%)	1 (100%)	1 (100%)
Sputum (*n* = 1)	NA	NA	1 (100%)	Invalid	NA
Unspecified (*n* = 1)	NA	NA	1 (100%)	1 (100%)	0 (0%)
Total (*n* = 70)	5 (7%)	19 (27%)	70 (100%)	68 (97%)	38 (54%)

^*a*^ NA, not available.

## DISCUSSION

Timely and accurate detection of Mucorales fungi from clinical specimens is crucial for improving patient outcomes by enabling the prompt initiation of targeted antifungal therapy (20, 21). Traditional diagnostic methods, such as culture and microscopy, face significant challenges. Culture methods require stringent processing and storage conditions to preserve hyphal viability, while histopathological diagnosis requires considerable expertise, cannot assign species-level identification, and is often made late in the course of infection, limiting its utility for guiding timely therapeutic interventions. In rapidly progressive mucormycosis, such delays can negatively impact patient outcomes. Additionally, a retrospective review by Kung et al*.* highlighted frequent misdiagnoses of *Aspergillus* spp. and Mucorales on histopathological examination (22). Promisingly, numerous in-house and commercial Mucorales PCR-based assays have been described (12, 18, 19, 23, 24) and show significant potential in expediting the diagnosis of mucormycosis (14, 25–27).

The newly developed Pan-Mucorales PCR demonstrated excellent performance, with 98.6% sensitivity and 100% specificity when evaluated on a variety of clinical specimens, including fresh and FFPE tissues, fluid, BALF/BW, CSF, and bone. Among currently available commercial PCR assays, MucorGenius (PathoNostics, Netherlands) has undergone the most clinical evaluation, primarily using serum samples, which were not assessed in this study. Guegan et al*.* evaluated the performance of the MucorGenius assay on pulmonary specimens (including BALF, tracheal aspirates, sputum, pleural fluids, and lung biopsies) and reported 90% sensitivity and 97.9% specificity in patients with proven/probable invasive mucormycosis (26) as defined by the European Organization for Research and Treatment of Cancer/Mycosis Study Group Education and Research Consortium (EORTC-MSGERC) guidelines (9). On the other hand, Rafanomezantsoa et al*.* compared the performance of MycoGENIE *Aspergillus-Mucorales* spp. PCR Kit (Ademtech, Pessac, France) and Fungiplex *Mucorales* RUO Real-Time PCR Kit (Bruker, Bremen, Germany) using a range of positive clinical samples (including blood, skin, respiratory, sinus, CSF, and other samples) (14) detected on an in-house real-time PCR assay based on methodology described and validated by Millon et al. (23). The MycoGENIE kit showed better agreement with the in-house assay, detecting 83% of samples with detectable Mucorales DNA, while the Fungiplex assay detected only 51% (14). Additionally, the MycoGENIE kit demonstrated higher sensitivity (88%) for detecting combined *Rhizopus* spp./*Mucor* spp., compared with the Fungiplex assay (44%) (14). Comparatively, our Pan-Mucorales PCR demonstrated superior sensitivity, likely due to its streamlined design of only targeting two sequences (18S rDNA and HBG), which reduces competitive inhibition and improves sensitivity. Furthermore, the high specificity of the assay may be attributed to the considered selection of primers and probes to minimize cross-reactivity, combined with optimization of annealing temperature during assay development.

The multiplex PCR assay, used as a follow-up to the Pan-Mucorales PCR, achieved genus/species-level identification with a sensitivity of 93.8% for *R. arrhizus*, 70.8% for *R. microsporus*, and 75% for *Mucor* spp. Discrepant (false negative) results for *R. arrhizus* (*n* = 1) and *R. microsporus* (*n* = 7) PCR targets were all linked to specimens with low fungal DNA, as indicated by high C_p_ values >30 on the Pan-Mucorales PCR. Notably, the assay was unable to detect *Mucor amphibiorum*, a species pathogenic to amphibians and platypuses but not reported to cause human infection (28). Although the two discordant specimens were collected from human patients, the clinical significance of these *M. amphibiorum* results remains unclear. Aside from this exception, the assay successfully detected all other *Mucor* spp. known to be pathogenic to humans (*n* = 10). The assay’s inability to detect *M. amphibiorum* is likely due to genomic variations affecting primer/probe binding sites, preventing PCR amplification. This is supported by the lack of detection of DNA from both cultures and clinical samples containing *M. amphibiorum,* confirmed by ITS sequencing. Furthermore, we observed excellent specificity (100%) for all three targets on this assay, which may be in part due to the selection of primers and probes targeting the ITS region, which offers favorable intra- and inter-species variability (29). A key advantage of this multiplex assay is its ability to provide fast genus/species-level identification for the three most frequently encountered Mucorales pathogens in Australia, without the need for post-PCR sequencing. As these three species account for approximately 63.6% of mucormycosis cases in Australia (2), the assay is expected to cover a significant proportion of cases. In comparison to commercially available Mucorales PCR assays, which do not provide genus or species-level identification (14, 15), our assay offers additional information for epidemiological surveillance and possibly for outbreak investigation (15). Notably, specimens that test positive on the Pan-Mucorales PCR but negative on the multiplex assay can undergo reflex testing using the existing panfungal PCR for further identification. Alternatively, the Pan-Mucorales PCR amplicons may be sequenced; however, species-level identification may not be reliably achieved due to the small fragment size (90–100 bp).

Our current molecular method for the diagnosis of mucormycosis is a panfungal PCR with DNA sequencing, which has an average TAT of 7 days for a positive result. In contrast, our proposed algorithm, combining Pan-Mucorales PCR with a multiplex genus/species-specific PCR, offers a significantly faster alternative. Pan-Mucorales PCR results can be obtained within 24–48 h of sample receipt for fresh specimens, with the assay itself taking 3 h. This rapid turnaround is crucial for the early initiation of first-line anti-Mucorales therapy, liposomal amphotericin B (15). The multiplex assay to further identify *R. arrhizus*, *R. microsporus*, and *Mucor* spp. only requires an additional 4–5 h. However, this additional time required for genus/species level identification should not delay the initiation of appropriate Mucorales targeted therapy, as the Pan-Mucorales PCR provides sufficient information to start treatment. For FFPE specimens, a longer TAT of 3–5 days is expected due to the more labor-intensive extraction method utilized. Lastly, for the identification of rare Mucorales species, the TAT is comparable to the panfungal PCR, though these species are relatively uncommon in Australia (2).

A key strength of the new assays is their versatility. They can be used across a wide range of clinical specimens from sterile and non-sterile sites and are particularly valuable for diagnosing proven and probable invasive mucormycosis, as defined by EORTC-MSGERC guidelines (9). Additionally, there may be potential to integrate the Pan-Mucorales PCR with the existing in-house *Aspergillus* PCR in our laboratory to improve detection of co-infections, which can occur in 30%–45% of cases (30, 31). A combined assay would enhance therapeutic guidance, particularly as first-line treatment for aspergillosis, voriconazole, is ineffective against mucormycosis. At present, clinical samples that test positive on the *Aspergillus* PCR are not subjected to the panfungal PCR, potentially missing co-infections. Further validation of this combined assay on non-invasive sample types, such as serum, could expand its application to screening and surveillance in high-risk, immunocompromised populations. There is substantial evidence supporting the use of such assays for the early detection of invasive mucormycosis and for monitoring therapeutic response in these patients (23, 25, 32).

From a cost perspective, the high setup and ongoing costs associated with commercially available Mucorales PCR assays have limited their routine adoption in many laboratories. Additionally, none are currently approved by the Therapeutic Goods Administration as *in vitro* diagnostic medical devices. Our proposed two-assay testing algorithm is significantly more economical, costing approximately $35 AUD per sample, with an additional $15 AUD for the multiplex assay. This also compares favourably to the $100 AUD cost of panfungal PCR with sequencing, making the new workflow more accessible for routine use. Furthermore, a combined in-house Pan-Mucorales/*Aspergillus* PCR is estimated to cost only marginally more than the current *Aspergillus* PCR alone.

This study has several limitations. First, panfungal PCR was used as the gold standard comparator, as the molecular reference for Mucorales diagnosis in our laboratory. This may lead to an overestimation of the sensitivity and specificity of the developed assays. Additionally, analytical and clinical studies comparing the performance of the Pan-Mucorales and targeted PCR assays against traditional methods would offer further insight into their diagnostic utility. Second, the multiplex targeted PCR assay includes only three genus/species-specific targets, excluding the internal control (HBG), due to the LightCycler 480 II instrument being limited to four fluorescence channels. While transitioning the assay to another instrument could allow for the inclusion of additional targets, increasing the number of PCR targets may reduce sensitivity, and a more practical approach would be to develop a second targeted PCR assay that includes additional Mucorales targets such as *Lichtheimia corymbifera*, *Rhizomucor* spp., and *Apophysomyces* spp. Lastly, the multiplex targeted PCR is unable to detect *M. amphibiorum,* a rare Mucorales of unclear clinical significance in humans. However, as it is detectable via the Pan-Mucorales PCR, our proposed diagnostic workflow reflexes the DNA extract to panfungal PCR with ITS sequencing for species-level identification if the targeted PCR assays are negative. Despite these limitations, the multiplex targeted PCR provides useful information for diagnosis, as well as for epidemiological surveillance and outbreak investigation. The Pan-Mucorales PCR offers sufficient information to initiate treatment for mucormycosis with liposomal amphotericin B, with isavuconazole or posaconazole as alternatives for patients with pre-existing renal impairment (15).

In conclusion, the streamlined diagnostic workflow consisting of two sequential real-time PCR assays presented here demonstrates excellent potential for improving mucormycosis diagnosis by offering rapid, accurate, and cost-effective detection of Mucorales pathogens. This approach not only facilitates the timely initiation of appropriate antifungal therapy to optimize patient outcomes but also provides valuable data for epidemiological surveillance and outbreak investigations.

## SUPPLEMENTAL MATERIAL

Supplemental material jcm.01937-24-s0001.pdf Includes Appendix 1 for analytical sensitivity and specificity of Pan-Mucorales PCR, Appendix 2 for analytical sensitivity and specificity of multiplex genus/species-specific PCR, Appendix 3 for testing on clinical samples, and Appendix 4 for limit of detection.

ASM does not own the copyrights to Supplemental Material that may be linked to, or accessed through, an article. The authors have granted ASM a non-exclusive, world-wide license to publish the Supplemental Material files. Please contact the corresponding author directly for reuse.
