# Supplementary material for: Development and evaluation of a Pan-Mucorales Real-time PCR and a multiplex Real-time PCR for detection and identification of Rhizopus arrhizus, Rhizopus microsporus, and Mucor spp. in clinical specimens
Source: J Clin Microbiol. 2025 Apr 30;63(6):e01937-24. doi: 10.1128/jcm.01937-24 (PMC12153301; doi:10.1128/jcm.01937-24)
Supplement: Supplemental material — Includes Appendix 1 for analytical sensitivity and specificity of Pan-Mucorales PCR, Appendix 2 for analytical sensitivity and specificity of multiplex genus/species-specific PCR, Appendix 3 for testing on clinical samples, and Appendix 4 for limit of detection. [file jcm.01937-24-s0001.pdf]

Supplementary Materials

Appendix 1

Analytical Sensitivity and Specificity of Pan-Mucorales PCR

Pan-Mucorales PCR testing on Mucorales fungal culture isolates

**No.**	**Organism**	**Pan-Mucorales PCR C_p**	**Pan-Mucorales PCR interpretation**	**Concordance**
1	*Rhizopus arrhizus*	19.49	Detected	Yes
2	*Rhizopus arrhizus*	20.43	Detected	Yes
3	*Rhizopus arrhizus*	19.69	Detected	Yes
4	*Rhizopus arrhizus*	20.33	Detected	Yes
5	*Rhizopus arrhizus*	21.96	Detected	Yes
6	*Rhizopus arrhizus*	20.23	Detected	Yes
7	*Rhizopus arrhizus*	19.32	Detected	Yes
8	*Rhizopus arrhizus*	19.14	Detected	Yes
9	*Rhizopus arrhizus*	19.09	Detected	Yes
10	*Rhizopus arrhizus*	18.91	Detected	Yes
11	*Rhizopus arrhizus*	22.79	Detected	Yes
12	*Rhizopus microsporus*	19.77	Detected	Yes
13	*Rhizopus microsporus*	19.12	Detected	Yes
14	*Rhizopus microsporus*	21.72	Detected	Yes
15	*Rhizopus microsporus*	17.84	Detected	Yes
16	*Rhizopus microsporus*	18.59	Detected	Yes
17	*Rhizopus microsporus*	18.27	Detected	Yes
18	*Rhizopus microsporus*	17.89	Detected	Yes
19	*Rhizopus microsporus*	19.83	Detected	Yes
20	*Rhizopus microsporus*	17.93	Detected	Yes
21	*Rhizopus microsporus*	19.22	Detected	Yes
22	*Rhizopus microsporus*	17.50	Detected	Yes
23	*Rhizopus microsporus*	18.27	Detected	Yes
24	*Mucor ramosussimus (circinelloides* complex*)*	21.59	Detected	Yes
25	*Mucor circinelloides*	19.32	Detected	Yes
26	*Mucor circinelloides*	24.24	Detected	Yes
27	*Mucor circinelloides*	19.71	Detected	Yes
28	*Mucor circinelloides*	20.01	Detected	Yes
29	*Mucor spp.*	19.91	Detected	Yes
30	*Mucor circinelloides*	33.55	Detected	Yes
31	*Mucor circinelloides*	18.63	Detected	Yes
32	*Mucor circinelloides*	19.91	Detected	Yes
33	*Mucor amphibiorum*	17.72	Detected	Yes
34	*Mucor irregularis (prev. Rhizomucor variabilis)*	16.65	Detected	Yes
35	*Rhizomucor pusillus*	21.06	Detected	Yes
36	*Rhizomucor miehei*	23.15	Detected	Yes
37	*Cunninghamella spp.* (most closely resembling *Cunninghamella multiverticilata)*	24.30	Detected	Yes
38	*Cunninghamella bertholletiae*	23.54	Detected	Yes
39	*Syncephalastrum racemosum*	23.74	Detected	Yes
40	*Syncephalastrum racemosum*	18.36	Detected	Yes
41	*Syncephalastrum racemosum*	20.71	Detected	Yes
42	*Syncephalastrum racemosum*	19.81	Detected	Yes

43	*Syncephalastrum racemosum*	21.13	Detected	Yes
44	*Saksenaea vasiformis* complex	17.22	Detected	Yes
45	*Saksenaea vasiformis* complex	21.79	Detected	Yes
46	*Lichtheimia ramosa*	19.82	Detected	Yes
47	*Lichtheimia ramosa*	19.58	Detected	Yes
48	*Lichtheimia ramosa*	17.89	Detected	Yes
49	*Lichtheimia ramosa*	21.88	Detected	Yes
50	*Lichtheimia ramosa*	35.51	Detected	Yes
51	*Lichtheimia corymbifera*	21.16	Detected	Yes
52	*Lichtheimia corymbifera*	19.13	Detected	Yes
53	*Lichtheimia corymbifera*	19.69	Detected	Yes
54	*Lichtheimia corymbifera*	20.15	Detected	Yes
55	*Lichtheimia corymbifera*	19.00	Detected	Yes
56	*Lichtheimia corymbifera*	19.81	Detected	Yes

C_p = crossing point; PCR = polymerase chain reaction.

Pan-Mucorales PCR testing on non-Mucorales fungal culture isolates

**No.**	**Organism**	**Pan-Mucorales PCR Interpretation**	**Concordance**
1	*Alternaria spp.*	Not detected	Yes
2	*Aspergillus calidoustus*	Not detected	Yes
3	*Aspergillus flavus* complex	Not detected	Yes
4	*Aspergillus fumigatus*	Not detected	Yes
5	*Aspergillus nidulans* complex	Not detected	Yes
6	*Aspergillus niger* complex	Not detected	Yes
7	*Candida albicans*	Not detected	Yes
8	*Candida albicans*	Not detected	Yes
9	*Cladophialophora bantiana*	Not detected	Yes
10	*Conidiobolus spp.*	Not detected	Yes
11	*Cryptococcus gattii*	Not detected	Yes
12	*Cryptococcus neoformans* var. *grubii*	Not detected	Yes
13	*Exophiala dermatitidis*	Not detected	Yes
14	*Exophiala oligosperma*	Not detected	Yes
15	*Fusarium oxysporum* complex	Not detected	Yes
16	*Fusarium solani* complex	Not detected	Yes
17	*Lomentospora prolificans*	Not detected	Yes
18	*Nakaseomyces glabratus*	Not detected	Yes
19	*Penicillium spp.*	Not detected	Yes
20	*Scedosporium apiospermum*	Not detected	Yes
21	*Scedosporium apiospermum*	Not detected	Yes
22	*Scedosporium aurantiacum*	Not detected	Yes
23	*Scedosporium boydii*	Not detected	Yes
24	*Talaromyces spp.* (not *marneffei*)	Not detected	Yes

C_p = crossing point; PCR = polymerase chain reaction; QC strain = quality control strain.

Pan-Mucorales PCR testing on clinical sample containing Pneumocystis jirovecii DNA (PCR-positive)

**Accession**	**Sample type**	**PCP PCR**	**Pan-Mucorales PCR**		**Concordance**
			**Pan-Mucorales PCR C_p**	**HBG C_p**	

**02-24-136-1416**	BAL	Detected	Not detected	26.31	Yes

BAL = bronchoalveolar lavage; C_p = crossing point; PCP = *Pneumocystis jirovecii;* PCR = polymerase chain reaction.

Pan-Mucorales PCR testing on bacterial culture isolates

No.	Organism	Pan-Mucorales PCR Interpretation	Concordance
1	*Staphylococcus aureus*	Not detected	Yes
2	*Haemophilus influenzae*	Not detected	Yes
3	*Streptococcus pyogenes*	Not detected	Yes
4	*Streptococcus pneumoniae*	Not detected	Yes
5	*Streptococcus oralis/mitis*	Not detected	Yes
6	*Streptococcus dysgalactiae*	Not detected	Yes
7	*Escherichia coli*	Not detected	Yes
8	*Pseudomonas aeruginosa*	Not detected	Yes
9	*Klebsiella pneumoniae*	Not detected	Yes
10	*Moraxella catarrhalis*	Not detected	Yes

PCR = polymerase chain reaction

Appendix 2

Analytical Sensitivity and Specificity of Multiplex genus/species-specific PCR

Rhizopus arrhizus PCR target on multiplex genus/species-specific PCR

Rhizopus arrhizus PCR testing on R. arrhizus culture isolates

No.	Organism	***R. arrhizus*** C_p	***R. arrhizus*** **PCR Interpretation**	**Concordance**
1	*Rhizopus arrhizus*	20.30	Detected	Yes
2	*Rhizopus arrhizus*	20.60	Detected	Yes
3	*Rhizopus arrhizus*	21.41	Detected	Yes
4	*Rhizopus arrhizus*	22.13	Detected	Yes
5	*Rhizopus arrhizus*	24.44	Detected	Yes
6	*Rhizopus arrhizus*	21.88	Detected	Yes
7	*Rhizopus arrhizus*	20.98	Detected	Yes
8	*Rhizopus arrhizus*	20.98	Detected	Yes
9	*Rhizopus arrhizus*	22.05	Detected	Yes
10	*Rhizopus arrhizus*	20.52	Detected	Yes
11	*Rhizopus arrhizus*	25.39	Detected	Yes

C_p = crossing point; PCR = polymerase chain reaction.

Rhizopus arrhizus PCR testing on other Mucorales fungal culture isolates

No.	Organism	***R. arrhizus*** **PCR Interpretation**	**Concordance**
1	*Rhizopus microsporus*	Not detected	Yes
2	*Rhizopus microsporus*	Not detected	Yes
3	*Mucor ramosussimus (circinelloides complex)*	Not detected	Yes
4	*Mucor circinelloides*	Not detected	Yes
5	*Mucor irregularis (prev. Rhizomucor variabilis)*	Not detected	Yes
6	*Rhizomucor pusillus*	Not detected	Yes
7	*Rhizomucor miehei*	Not detected	Yes
8	*Cunninghamella spp.* (most closely resembling *Cunninghamella multiverticilata)*	Not detected	Yes
9	*Syncephalastrum racemosum*	Not detected	Yes
10	*Saksenaea vasiformis complex*	Not detected	Yes
11	*Lichtheimia ramosa*	Not detected	Yes
12	*Lichtheimia corymbifera*	Not detected	Yes
13	*Lichtheimia corymbifera*	Not detected	Yes
14	*Lichtheimia corymbifera*	Not detected	Yes
15	*Lichtheimia corymbifera*	Not detected	Yes
16	*Lichtheimia corymbifera*	Not detected	Yes

PCR = polymerase chain reaction

Rhizopus arrhizus PCR testing on non-Mucorales fungal culture isolates

No.	Organism	*R. arrhizus* PCR Interpretation	Concordance
1	*Alternaria spp.*	Not detected	Yes
2	*Aspergillus calidoustus*	Not detected	Yes
3	*Aspergillus flavus complex*	Not detected	Yes
4	*Aspergillus fumigatus*	Not detected	Yes
5	*Aspergillus nidulans complex*	Not detected	Yes
6	*Aspergillus niger complex*	Not detected	Yes
7	*Candida albicans*	Not detected	Yes
8	*Candida glabrata*	Not detected	Yes
9	*Cladophialophora bantiana*	Not detected	Yes
10	*Conidiobolus spp.*	Not detected	Yes
11	*Cryptococcus gattii*	Not detected	Yes
12	*Cryptococcus neoformans var grubii*	Not detected	Yes
13	*Exophiala dermatitidis*	Not detected	Yes
14	*Exophiala oligosperma*	Not detected	Yes
15	*Fusarium oxysporum complex*	Not detected	Yes
16	*Fusarium solani complex*	Not detected	Yes
17	*Lomentospora prolificans*	Not detected	Yes
18	*Penicillium spp.*	Not detected	Yes
19	*Scedosporium apiospermum*	Not detected	Yes
20	*Scedosporium apiospermum*	Not detected	Yes
21	*Scedosporium aurantiacum*	Not detected	Yes
22	*Scedosporium boydii*	Not detected	Yes
23	*Talaromyces spp. (not marneffei)*	Not detected	Yes

PCR = polymerase chain reaction; QC strain = quality control strain.

Rhizopus arrhizus PCR testing on bacterial culture isolates

No.	Organism	*R. arrhizus* PCR Interpretation	Concordance
1	*Staphylococcus aureus*	Not detected	Yes
2	*Haemophilus influenzae*	Not detected	Yes
3	*Streptococcus pyogenes*	Not detected	Yes
4	*Streptococcus pneumoniae*	Not detected	Yes
5	*Streptococcus oralis/mitis*	Not detected	Yes
6	*Streptococcus dysgalactiae*	Not detected	Yes
7	*Escherichia coli*	Not detected	Yes
8	*Pseudomonas aeruginosa*	Not detected	Yes
9	*Klebsiella pneumoniae*	Not detected	Yes
10	*Moraxella catarrhalis*	Not detected	Yes

PCR = polymerase chain reaction

Rhizopus microsporus PCR target on multiplex genus/species-specific PCR

Rhizopus microsporus PCR testing on Rhizopus microsporus culture isolates

**No.**	**Organism**	***R. microsporus*** **C_p**	***R. microsporus*** **PCR Interpretation**	**Concordance**
1	*Rhizopus microsporus*	22.32	Detected	Yes
2	*Rhizopus microsporus*	19 96	Detected	Yes
3	*Rhizopus microsporus*	24.35	Detected	Yes
4	*Rhizopus microsporus*	20.40	Detected	Yes
5	*Rhizopus microsporus*	21.19	Detected	Yes
6	*Rhizopus microsporus*	21.38	Detected	Yes
7	*Rhizopus microsporus*	20.94	Detected	Yes
8	*Rhizopus microsporus*	26.88	Detected	Yes
9	*Rhizopus microsporus*	21.02	Detected	Yes
10	*Rhizopus microsporus*	22.36	Detected	Yes
11	*Rhizopus microsporus*	20.76	Detected	Yes
12	*Rhizopus microsporus*	21.31	Detected	Yes

C_p = crossing point; PCR = polymerase chain reaction.

Rhizopus microsporus PCR testing on other Mucorales fungal culture isolates

**No.**	**Organism**	***R. microsporus*** **PCR Interpretation**	**Concordance**
1	*Rhizopus arrhizus*	Not detected	Yes
2	*Rhizopus arrhizus*	Not detected	Yes
3	*Mucor ramosussimus (circinelloides complex)*	Not detected	Yes
4	*Mucor circinelloides*	Not detected	Yes
5	*Mucor irregularis (prev. Rhizomucor variabilis)*	Not detected	Yes
6	*Rhizomucor pusillus*	Not detected	Yes
7	*Rhizomucor miehei*	Not detected	Yes
8	*Cunninghamella spp. (most resembling Cunninghamella multiverticilata)*	Not detected	Yes
9	*Syncephalastrum racemosum*	Not detected	Yes
10	*Saksenaea vasiformis complex*	Not detected	Yes
11	*Lichtheimia ramosa*	Not detected	Yes
12	*Lichtheimia corymbifera*	Not detected	Yes
13	*Lichtheimia corymbifera*	Not detected	Yes
14	*Lichtheimia corymbifera*	Not detected	Yes
15	*Lichtheimia corymbifera*	Not detected	Yes
16	*Lichtheimia corymbifera*	Not detected	Yes

PCR = polymerase chain reaction

Rhizopus microsporus PCR testing on non-Mucorales fungal culture isolates

No.	Organism	*R. microsporus* PCR Interpretation	Concordance
1	*Alternaria spp.*	Not detected	Yes
2	*Aspergillus calidoustus*	Not detected	Yes
3	*Aspergillus flavus complex*	Not detected	Yes
4	*Aspergillus fumigatus*	Not detected	Yes
5	*Aspergillus nidulans complex*	Not detected	Yes
6	*Aspergillus niger complex*	Not detected	Yes
7	*Candida albicans*	Not detected	Yes
8	*Candida glabrata*	Not detected	Yes
9	*Cladophialophora bantiana*	Not detected	Yes
10	*Conidiobolus spp.*	Not detected	Yes
11	*Cryptococcus gattii*	Not detected	Yes
12	*Cryptococcus neoformans var grubii*	Not detected	Yes
13	*Exophiala dermatitidis*	Not detected	Yes
14	*Exophiala oligosperma*	Not detected	Yes
15	*Fusarium oxysporum complex*	Not detected	Yes
16	*Fusarium solani complex*	Not detected	Yes
17	*Lomentospora prolificans*	Not detected	Yes
18	*Penicillium spp.*	Not detected	Yes
19	*Scedosporium apiospermum*	Not detected	Yes
20	*Scedosporium apiospermum*	Not detected	Yes
21	*Scedosporium aurantiacum*	Not detected	Yes
22	*Scedosporium boydii*	Not detected	Yes
23	*Talaromyces spp. (not marneffei)*	Not detected	Yes

PCR = polymerase chain reaction; QC strain = quality control strain.

Rhizopus microsporus PCR testing on bacterial culture isolates

No.	Organism	*R. microsporus* PCR Interpretation	Concordance
1	*Staphylococcus aureus*	Not detected	Yes
2	*Haemophilus influenzae*	Not detected	Yes
3	*Streptococcus pyogenes*	Not detected	Yes
4	*Streptococcus pneumoniae*	Not detected	Yes
5	*Streptococcus oralis/mitis*	Not detected	Yes
6	*Streptococcus dysgalactiae*	Not detected	Yes
7	*Escherichia coli*	Not detected	Yes
8	*Pseudomonas aeruginosa*	Not detected	Yes
9	*Klebsiella pneumoniae*	Not detected	Yes
10	*Moraxella catarrhalis*	Not detected	Yes

PCR = polymerase chain reaction

<u>*Mucor* spp. PCR target on multiplex genus/species-specific PCR</u>

Mucor spp. PCR testing on Mucor spp. culture isolates

No.	Organism	***Mucor* spp. C_p**	***Mucor* spp. PCR Interpretation**	**Concordance**
1	*Mucor ramosussimus (circinelloides complex)*	24.28	Detected	Yes
2	*Mucor circinelloides*	21.81	Detected	Yes
3	*Mucor circinelloides*	28.75	Detected	Yes
4	*Mucor circinelloides*	23.97	Detected	Yes
5	*Mucor circinelloides*	24.46	Detected	Yes
6	*Mucor spp.*	24.03	Detected	Yes
7	*Mucor circinelloides*	39.75	Detected	Yes
8	*Mucor circinelloides*	22.88	Detected	Yes
9	*Mucor circinelloides*	24.27	Detected	Yes
10	*Mucor amphibiorum*	**Not detected**	**Not detected**	**No**
11	*Mucor irregularis (prev. Rhizomucor variabilis)*	27.93	Detected	Yes

C_p = crossing point; PCR = polymerase chain reaction.

Mucor spp. PCR testing on other Mucorales fungal culture isolates

No.	Organism	***Mucor* spp. PCR Interpretation**	**Concordance**
1	*Rhizopus arrhizus*	Not detected	Yes
2	*Rhizopus arrhizus*	Not detected	Yes
3	*Rhizopus microsporus*	Not detected	Yes
4	*Rhizopus microsporus*	Not detected	Yes
5	*Rhizomucor pusillus*	Not detected	Yes
6	*Rhizomucor miehei*	Not detected	Yes
7	*Cunninghamella spp.* (most resembling *Cunninghamella multiverticilata*)	Not detected	Yes
8	*Syncephalastrum racemosum*	Not detected	Yes
9	*Syncephalastrum racemosum*	Not detected	Yes
10	*Syncephalastrum racemosum*	Not detected	Yes
11	*Syncephalastrum racemosum*	Not detected	Yes
12	*Saksenaea vasiformis complex*	Not detected	Yes
13	*Saksenaea vasiformis complex*	Not detected	Yes
14	*Lichtheimia ramosa*	Not detected	Yes
15	*Lichtheimia ramosa*	Not detected	Yes
16	*Lichtheimia corymbifera*	Not detected	Yes
17	*Lichtheimia corymbifera*	Not detected	Yes
18	*Lichtheimia corymbifera*	Not detected	Yes
19	*Lichtheimia corymbifera*	Not detected	Yes

PCR = polymerase chain reaction

Mucor spp. PCR testing on non-Mucorales fungal culture isolates

No.	Organism	*Mucor* spp. PCR Interpretation	Concordance
1	*Alternaria spp.*	Not detected	Yes
2	*Aspergillus calidoustus*	Not detected	Yes
3	*Aspergillus flavus complex*	Not detected	Yes
4	*Aspergillus fumigatus*	Not detected	Yes
5	*Aspergillus nidulans complex*	Not detected	Yes
6	*Aspergillus niger complex*	Not detected	Yes
7	*Candida albicans*	Not detected	Yes
8	*Candida glabrata*	Not detected	Yes
9	*Cladophialophora bantiana*	Not detected	Yes
10	*Conidiobolus spp.*	Not detected	Yes
11	*Cryptococcus gattii*	Not detected	Yes
12	*Cryptococcus neoformans var grubii*	Not detected	Yes
13	*Exophiala dermatitidis*	Not detected	Yes
14	*Exophiala oligosperma*	Not detected	Yes
15	*Fusarium oxysporum complex*	Not detected	Yes
16	*Fusarium solani complex*	Not detected	Yes
17	*Lomentospora prolificans*	Not detected	Yes
18	*Penicillium spp.*	Not detected	Yes
19	*Scedosporium apiospermum*	Not detected	Yes
20	*Scedosporium apiospermum*	Not detected	Yes
21	*Scedosporium aurantiacum*	Not detected	Yes
22	*Scedosporium boydii*	Not detected	Yes
23	*Talaromyces spp. (not marneffei)*	Not detected	Yes

PCR = polymerase chain reaction; QC strain = quality control strain.

Mucor spp. (Muc) PCR testing on bacterial culture isolates

No.	Organism	*Mucor* spp. PCR Interpretation	Concordance
1	*Staphylococcus aureus*	Not detected	Yes
2	*Haemophilus influenzae*	Not detected	Yes
3	*Streptococcus pyogenes*	Not detected	Yes
4	*Streptococcus pneumoniae*	Not detected	Yes
5	*Streptococcus oralis/mitis*	Not detected	Yes
6	*Streptococcus dysgalactiae*	Not detected	Yes
7	*Escherichia coli*	Not detected	Yes
8	*Pseudomonas aeruginosa*	Not detected	Yes
9	*Klebsiella pneumoniae*	Not detected	Yes
10	*Moraxella catarrhalis*	Not detected	Yes

PCR = polymerase chain reaction

Appendix 3

Testing on Clinical Samples

Pan-Mucorales (PanMuc18, HBG) PCR testing on positive clinical samples

	Sample type	Panfungal PCR result	Pan-Mucorales PCR		Concordance
			Pan-Mucorales target C_p	HBG target C_p	
1	Fresh tissue	*Apophysomyces variabilis*	28.80	31.93	Yes
2	FFPE tissue	*Apophysomyces variabilis*	27.20	24.43	Yes
3	Fresh tissue	*Apophysomyces variabilis*	24.48	28.22	Yes
4	FFPE tissue	*Cunninghamella bertholletiae*	24.10	29.14	Yes
5	Fresh tissue	*Cunninghamella bertholletiae*	24.48	Not detected	Yes
6	Fluid	*Cunninghamella bertholletiae*	30.16	28.55	Yes
7	Fresh tissue	*Cunninghamella bertholletiae*	23.25	32.07	Yes
8	Fluid	*Cunninghamella echinulata*	27.98	25.85	Yes
			29.01*	26.70*	Yes
9	Fluid	*Cunninghamella spp*	27.50	21.84	Yes
10	FFPE tissue	*Lichtheimia corymbifera*	22.27	32.43	Yes
11	Fresh tissue	*Mucor amphibiorum*	33.55	22.23	Yes
12	FFPE tissue	*Mucor amphibiorum*	26.47	28.31	Yes
			24.10*	25.87*	Yes
13	Fresh tissue	*Mucor circinelloides*	20.46	Not detected	Yes
14	Bone	*Mucor circinelloides*	26.83	25.47	Yes
15	Fresh tissue	*Mucor circinelloides*	8.3	Not detected	Yes
16	Sputum	*Mucor circinelloides*	**Not detected**	**28.14**	**No**
			**Not detected***	**27.00***	**No**
17	Fresh tissue	*Mucor circinelloides*	29.49	21.52	Yes
18	Fresh tissue	*Mucor circinelloides*	30.48*	18.81*	Yes

19	Fresh tissue	*Mucor hiemalis*	20.25*	Not detected (veterinary sample)*	Yes
20	Fluid	*Rhizomucor miehei*	29.38	25.32	Yes
21	Fresh tissue	*Rhizomucor pusillus*	29.35	28.12	Yes
22	Fresh tissue	*Rhizomucor pusillus*	33.85	29.73	Yes
23	Fresh tissue	*Rhizomucor pusillus*	28.93	23.77	Yes
24	BAL	*Rhizomucor pusillus*	31.56*	28.71*	Yes
25	FFPE tissue	*Rhizomucor pusillus*	**Not detected**	**Not detected**	**Invalid**
26	FFPE tissue	*Rhizomucor pusillus*	31.63	31.18	Yes
27	Fresh tissue	*Rhizomucor pusillus*	36.22*	30.91*	Yes
28	Fresh tissue	*Rhizopus arrhizus*	24.60	22.22	Yes
29	Fresh tissue	*Rhizopus arrhizus*	24.94	Not detected (veterinary sample)	Yes
30	Fresh tissue	*Rhizopus arrhizus*	20.90	Not detected (veterinary sample)	Yes
31	Fresh tissue	*Rhizopus arrhizus*	22.45	Not detected (veterinary sample)	Yes
32	Fresh tissue	*Rhizopus arrhizus*	22.32	24.78	Yes
33	BW	*Rhizopus arrhizus*	33.87	25.17	Yes
34	FFPE tissue	*Rhizopus arrhizus*	24.31	25.32	Yes
			25.18*	25.99*	Yes
35	FFPE tissue	*Rhizopus arrhizus*	27.82	29.47	Yes
36	Fresh tissue	*Rhizopus arrhizus*	26.52	25.93	Yes
37	CSF	*Rhizopus arrhizus*	33.36	27.59	Yes
38	Fresh tissue	*Rhizopus arrhizus*	35.02*	20.59*	Yes
39	CSF	*Rhizopus arrhizus*	33.87*	26.31*	Yes
40	FFPE tissue	*Rhizopus arrhizus*	28.11*	26.93*	Yes
41	BW	*Rhizopus arrhizus*	24.87*	24.16*	Yes
42	Fresh tissue	*Rhizopus arrhizus*	34.77*	30.39*	Yes
43	Fresh tissue	*Rhizopus arrhizus*	19.45	22.18	Yes
44	Fresh tissue	*Rhizopus microsporus*	32.62	19.93	Yes
45	BAL	*Rhizopus microsporus*	34.07	28.60	Yes
46	FFPE tissue	*Rhizopus microsporus*	23.70	Not detected (veterinary sample)	Yes

			24.53*	Not detected (veterinary sample)*	Yes
47	Fresh tissue	*Rhizopus microsporus*	28.07	22.98	Yes
48	FFPE tissue	*Rhizopus microsporus*	27.44	30.54	Yes
49	Fresh tissue	*Rhizopus microsporus*	22.63	21.61	Yes
50	Fresh tissue	*Rhizopus microsporus*	20.47	36.70	Yes
51	FFPE tissue	*Rhizopus microsporus*	21.58	27.44	Yes
52	Fluid	*Rhizopus microsporus*	28.06	25.32	Yes
53	FFPE tissue	*Rhizopus microsporus*	25.06	28.08	Yes
54	Fluid	*Rhizopus microsporus*	28.74	27.03	Yes
55	Fresh tissue	*Rhizopus microsporus*	33.98	30.04	Yes
56	FNA lung	*Rhizopus microsporus*	31.80*	25.23*	Yes
57	BW	*Rhizopus microsporus*	32.20	25.33	Yes
58	Fresh tissue	*Rhizopus microsporus*	32.71*	24.35*	Yes
59	Fresh tissue	*Rhizopus microsporus*	21.73*	24.08*	Yes
60	Fresh tissue	*Rhizopus microsporus*	24.6	32.04	Yes
61	Fresh tissue	*Rhizopus microsporus*	26.62	27.89	Yes
62	FFPE tissue	*Rhizopus microsporus*	23.92	Not detected (veterinary sample)	Yes
63	Fresh tissue	*Rhizopus microsporus*	22.58	21.61	Yes
64	Fresh tissue	*Rhizopus microsporus*	26.83	29.40	Yes
			25.54*	28.05*	Yes
65	Unspecified	*Rhizopus microsporus*	33.72*	21.34*	Yes
66	Fresh tissue	*Rhizopus microsporus*	33.25*	36.02*	Yes
67	Fresh tissue	*Rhizopus microsporus*	28.69*	29.81*	Yes
68	Fresh tissue	*Sakenaea vasiformis cx*	28.55	30.00	Yes
69	Fresh tissue	*Sakenaea vasiformis cx*	20.57	20.69	Yes
70	BW	*Syncephalastrum racemosum*	24.73	29.93	Yes

BAL = bronchioalveolar lavage; BW = bronchial washing; C_p = crossing point; CSF = cerebrospinal fluid; FFPE = formalin fixed paraffin embedded; FNA = fine needle aspirate; HBG = human β globin; PCR = polymerase chain reaction; * indicates new DNA extraction performed on archived clinical specimens.

Pan-Mucorales (PanMuc18, HBG) PCR testing on negative clinical samples

	Sample type	Panfungal PCR result	Pan-Mucorales PCR		Concordance
			PanMuc18 C_p	HBG C_p	
1	BW	Not detected	Not detected	22.49	Yes
2	FFPE tissue	Not detected	Not detected	32.84	Yes
3	BAL	Not detected	Not detected	32.97	Yes
4	Fresh tissue	Not detected	Not detected	18.42	Yes
5	Fluid	Not detected	Not detected	18.84	Yes
6	BW	Not detected	Not detected	24.79	Yes
7	Fresh tissue	Not detected	Not detected	20.61	Yes
8	Fluid	Not detected	Not detected	22.51	Yes
9	Fluid	Not detected	Not detected	28.92	Yes
10	BAL	Not detected	Not detected	22.6	Yes
11	Fluid	Not detected	Not detected	19.02	Yes
12	BAL	Not detected	Not detected	28.17	Yes
13	BW	*Candida albicans*	Not detected	20.59	Yes
14	FFPE tissue	*Candida parapsilosis*	Not detected	33.08	Yes
15	Fresh tissue	Not detected	Not detected	27.41	Yes
16	Fluid	Not detected	Not detected	20.35	Yes
17	Fresh tissue	Not detected	Not detected	31.43	Yes
18	BAL	Not detected	Not detected	24.18	Yes
19	BW	Not detected	Not detected	24.73	Yes
20	BAL	Not detected	Not detected	27.33	Yes
21	BW	*Candida albicans*	Not detected	28.38	Yes
22	Fresh tissue	Not detected	Not detected	28.69	Yes
23	Fluid	Not detected	Not detected	22.51	Yes
24	Fresh tissue	Not detected	Not detected	20.59	Yes
25	CSF	Not detected	Not detected	25.5	Yes
26	Fresh tissue	Not detected	Not detected	22.42	Yes
27	Fluid	Not detected	Not detected	26.84	Yes
28	Fresh tissue	Not detected	Not detected	24.2	Yes
29	Fluid	Not detected	Not detected	27.83	Yes
30	Fresh tissue	Not detected	Not detected	35.31	Yes

31	Fresh tissue	Not detected	Not detected	27.72	Yes
32	Fluid	Not detected	Not detected	20.87	Yes
33	Fresh tissue	Not detected	Not detected	26.49	Yes
34	BW	Not detected	Not detected	23.68	Yes
35	FNA	Not detected	Not detected	29.52	Yes
36	Bone marrow	Not detected	Not detected	24.53	Yes
37	BAL	Not detected	Not detected	22.48	Yes
38	BAL	Not detected	Not detected	19.66	Yes
39	Fresh tissue	Not detected	Not detected	21.91	Yes
40	BAL	Not detected	Not detected	21.92	Yes
41	BW	Not detected	Not detected	23.17	Yes
42	BW	Not detected	Not detected	19.66	Yes
43	BAL	Not detected	Not detected	24.12	Yes
44	BAL	Not detected	Not detected	24.2	Yes
45	Fluid	Not detected	Not detected	36.3	Yes
46	Fresh tissue	Not detected	Not detected	29.31	Yes
47	CSF	Not detected	Not detected	35.94	Yes
48	Fresh tissue	Not detected	Not detected	33.17	Yes
49	BAL	Not detected	Not detected	30.69	Yes
50	Fluid	Not detected	Not detected	22.41	Yes
51	Fresh tissue	Not detected	Not detected	23.9	Yes
52	FFPE tissue	Not detected	Not detected	34.3	Yes
53	BAL	Not detected	Not detected	25.22	Yes
54	Fresh tissue	Not detected	Not detected	18.72	Yes
55	Fresh tissue	Not detected	Not detected	24.95	Yes
56	Fresh tissue	Not detected	Not detected	28.07	Yes
57	Bone	Not detected	Not detected	19.89	Yes
58	BAL	Not detected	Not detected	25.73	Yes
59	Fluid	Not detected	Not detected	21.51	Yes
60	Fresh tissue	Not detected	Not detected	28.68	Yes
61	Fluid	*Aspergillus versicolor*	Not detected	27.22	Yes
62	Fresh tissue	*Aspergillus fumigatus*	Not detected	25.97	Yes
63	CSF	*Aspergillus penicillioides*	Not detected	33.61	Yes
64	BAL	*Aspergillus fumigatus*	Not detected	20.6	Yes
65	FFPE tissue	*Aspergillus nidulans*	Not detected	33.15	Yes

66	Fresh tissue	*Aspergillus fumigatus*	Not detected	26.47	Yes
67	Fresh tissue	*Aspergillus terreus*	Not detected	22.65	Yes
68	CSF	*Aspergillus fumigatus*	Not detected	30.84	Yes
69	FFPE tissue	*Aspergillus flavus*	Not detected	33.35	Yes
70	Fresh tissue	*Aspergillus flavus*	Not detected	25.58	Yes
71	Fresh tissue	*Aspergillus flavus*	Not detected	24.92	Yes
72	Fresh tissue	*Aspergillus fumigatus*	Not detected	24.32	Yes
73	Fresh tissue	*Aspergillus fumigatus*	Not detected	20.33	Yes
74	Fresh tissue	*Aspergillus fumigatus*	Not detected	24.65	Yes
75	Bone	*Aspergillus fumigatus*	Not detected	30.99	Yes
76	Fluid	*Fusarium solani complex*	Not detected	20.9	Yes
77	Fluid	*Fusarium solani complex*	Not detected	18.62	Yes
78	Fresh tissue	*Fusarium solani complex*	Not detected	20.94	Yes
79	Fresh tissue	*Fusarium solani complex*	Not detected	19.57	Yes
80	FFPE tissue	*Fusarium solani complex*	Not detected	20.71	Yes
81	Fresh tissue	*Fusarium solani complex*	Not detected	25.95	Yes
82	Fluid	*Meyerozyma guilliermondii*	Not detected	27	Yes
83	Fresh tissue	*Candida famata*	Not detected	20.61	Yes
84	Fluid	*Trichosporon inkin*	Not detected	28.69	Yes
85	Fluid	*Scedosporium boydii*	Not detected	27.98	Yes
86	Fluid	*Lomentospora prolificans*	Not detected	31.86	Yes
87	Fluid	*Scedosporium apiospermum*	Not detected	26.4	Yes
88	Fluid	*Scedosporium boydii*	Not detected	19.69	Yes
89	FFPE tissue	*Scedosporium apiospermum*	Not detected	29.01	Yes
90	Fluid	*Scedosporium boydii*	Not detected	20.63	Yes
91	Fresh tissue	*Scedosporium apiospermum*	Not detected	17.92	Yes
92	Fresh tissue	*Pseudallescheria boydii*	Not detected	32.15	Yes
93	FFPE tissue	*Trichophyton rubrum*	Not detected	32.47	Yes
94	Fresh tissue	*Microsphaeropsis aerundinis*	Not detected	28.74	Yes
95	Fluid	*Exserohilum rostratum*	Not detected	25.48	Yes

BAL = bronchioalveolar lavage; BW = bronchial washing; C_p = crossing point; CSF = cerebrospinal fluid; FFPE = formalin fixed paraffin embedded; FNA = fine needle aspirate; HBG = human β globin; PCR = polymerase chain reaction.

Multiplex genus/species-specific PCR testing on positive clinical samples

	Sample type	Panfungal PCR result	Multiplex genus/species-specific PCR				Interpretation	Concordance
			***R. arrhizus* PCR C_p**	***R. microsporus* PCR C_p**	***Mucor* spp. PCR C_p**	**HBG C_p**		
1	Fresh tissue	*Mucor amphibiorum*	Not detected	Not detected	Not detected	22.05	**Not detected**	**No**
2	FFPE tissue	*Mucor amphibiorum*	Not detected	Not detected	Not detected	30.68	**Not detected**	**No**
			Not detected*	Not detected*	Not detected*	28.22*	**Not detected**	**No**
3	Fresh tissue	*Mucor circinelloides*	Not detected	Not detected	22.61	ND	*Mucor* spp. DNA detected	Yes
4	Bone	*Mucor circinelloides*	Not detected	Not detected	31.01	25.79	*Mucor* spp. DNA detected	Yes
5	Fresh tissue	*Mucor circinelloides*	Not detected	Not detected	13.6	ND	*Mucor* spp. DNA detected	Yes
6	Fresh tissue	*Mucor circinelloides*	Not detected	Not detected	33.36	21.66	*Mucor* spp. DNA detected	Yes
7	Fresh tissue	*Mucor circinelloides*	Not detected*	Not detected*	34.15*	19.18*	*Mucor* spp. DNA detected	Yes
8	Fresh tissue	*Mucor hiemalis*	Not detected*	Not detected*	31.60*	Not detected (veterinary sample)*	*Mucor* spp. DNA detected	Yes
9	Fresh tissue	*Rhizopus arrhizus*	25.28	Not detected	Not detected	22.08	*R. arrhizus* DNA detected	Yes
10	Fresh tissue	*Rhizopus arrhizus*	26.66	Not detected	Not detected	Not detected (veterinary sample)	*R. arrhizus* DNA detected	Yes
11	Fresh tissue	*Rhizopus arrhizus*	22.80	Not detected	Not detected	Not detected (veterinary sample)	*R. arrhizus* DNA detected	Yes
12	Fresh tissue	*Rhizopus arrhizus*	24.39	Not detected	Not detected	Not detected (veterinary sample)	*R. arrhizus* DNA detected	Yes
13	Fresh tissue	*Rhizopus arrhizus*	24.56	Not detected	Not detected	25.7	*R. arrhizus* DNA detected	Yes
14	BW	*Rhizopus arrhizus*	33.6	Not detected	Not detected	25.3	*R. arrhizus* DNA detected	Yes
15	FFPE tissue	*Rhizopus arrhizus*	27.40*	Not detected*	Not detected*	27.08*	*R. arrhizus* DNA detected	Yes
16	FFPE tissue	*Rhizopus arrhizus*	36.55	Not detected	Not detected	37.27	*R. arrhizus* DNA detected	Yes
17	Fresh tissue	*Rhizopus arrhizus*	28.46	Not detected	Not detected	27.08	*R. arrhizus* DNA detected	Yes
18	CSF	*Rhizopus arrhizus*	Detected, Cp~35 but not recorded	Not detected	Not detected	27.7	*R. arrhizus* DNA detected	Yes
19	Fresh tissue	*Rhizopus arrhizus*	Not detected*	Not detected*	Not detected*	20.75*	**Not detected**	**No**
20	CSF	*Rhizopus arrhizus*	33.59*	Not detected*	Not detected*	26.78*	*R. arrhizus* DNA detected	Yes
21	FFPE tissue	*Rhizopus arrhizus*	28.50*	Not detected*	Not detected*	27.02*	*R. arrhizus* DNA detected	Yes

22	BW	*Rhizopus arrhizus*	26.28*	Not detected*	Not detected*	24.45*	*R. arrhizus* DNA detected	Yes
23	Fresh tissue	*Rhizopus arrhizus*	31.48*	Not detected*	Not detected*	29.20*	*R. arrhizus* DNA detected	Yes
24	Fresh tissue	*Rhizopus arrhizus*	21.04	Not detected	Not detected	22.67	*R. arrhizus* DNA detected	Yes
25	Fresh tissue	*Rhizopus microsporus*	Not detected	Not detected	Not detected	19.85	**Not detected**	**No**
26	BAL	*Rhizopus microsporus*	Not detected	Not detected	Not detected	28.7	**Not detected**	**No**
27	FFPE tissue	*Rhizopus microsporus*	Not detected	27.39	Not detected	Not detected (veterinary sample)	*R. microsporus* DNA detected	Yes
			Not detected*	28.18*	Not detected*	Not detected (veterinary sample)*	*R. microsporus* DNA detected	Yes
28	Fresh tissue	*Rhizopus microsporus*	Not detected	27.77	Not detected	22.96	*R. microsporus* DNA detected	Yes
29	FFPE tissue	*Rhizopus microsporus*	Not detected	30.99	Not detected	32	*R. microsporus* DNA detected	Yes
30	Fresh tissue	*Rhizopus microsporus*	Not detected	26.56	Not detected	22.67	*R. microsporus* DNA detected	Yes
31	Fresh tissue	*Rhizopus microsporus*	Not detected	24.35	Not detected	29.2	*R. microsporus* DNA detected	Yes
32	FFPE tissue	*Rhizopus microsporus*	Not detected	25.04	Not detected	29.24	*R. microsporus* DNA detected	Yes
33	Fluid	*Rhizopus microsporus*	Not detected	29.3	Not detected	25.47	*R. microsporus* DNA detected	Yes
34	FFPE tissue	*Rhizopus microsporus*	Not detected	30.26	Not detected	30.18	*R. microsporus* DNA detected	Yes
35	Fluid	*Rhizopus microsporus*	Not detected	30.05	Not detected	27.41	*R. microsporus* DNA detected	Yes
36	Fresh tissue	*Rhizopus microsporus*	Not detected	Not detected	Not detected	30.21	**Not detected**	**No**
37	FNA	*Rhizopus microsporus*	Not detected*	Not detected	Not detected*	25.45*	**Not detected**	**No**
38	BW	*Rhizopus microsporus*	Not detected	Not detected	Not detected	25.51	**Not detected**	**No**
39	Fresh tissue	*Rhizopus microsporus*	Not detected*	Not detected*	Not detected*	24.59*	**Not detected**	**No**

40	Fresh tissue	*Rhizopus microsporus*	Not detected*	27.84*	Not detected*	26.50*	*R. microsporus* DNA detected	Yes
41	Fresh tissue	*Rhizopus microsporus*	Not detected	27.33	Not detected	34.06	*R. microsporus* DNA detected	Yes
42	Fresh tissue	*Rhizopus microsporus*	Not detected	28.95	Not detected	29.16	*R. microsporus* DNA detected	Yes
43	FFPE tissue	*Rhizopus microsporus*	Not detected	28.2	Not detected	38.51	*R. microsporus* DNA detected	Yes
44	Fresh tissue	*Rhizopus microsporus*	Not detected	26.21	Not detected	22.78	*R. microsporus* DNA detected	Yes
45	Fresh tissue	*Rhizopus microsporus*	Not detected*	28.31*	Not detected*	30.26*	*R. microsporus* DNA detected	Yes
46	Unspecified	*Rhizopus microsporus*	Not detected*	Not detected*	Not detected*	21.02*	**Not detected**	**No**
47	Fresh tissue	*Rhizopus microsporus*	Not detected*	36.69*	Not detected*	37.29*	*R. microsporus* DNA detected	Yes
48	Fresh tissue	*Rhizopus microsporus*	Not detected*	30.86*	Not detected*	30.87*	*R. microsporus* DNA detected	Yes

BAL = bronchioalveolar lavage; BW = bronchial washing; C_p = crossing point; CSF = cerebrospinal fluid; FFPE = formalin fixed paraffin embedded; FNA = fine needle aspirate; HBG = human β globin; PCR = polymerase chain reaction; * indicates new DNA extraction performed on archived clinical specimens.

Multiplex genus/species-specific PCR testing on negative clinical samples

	Sample type	Panfungal PCR result	Multiplex genus/species-specific PCR				Concordance
			***R. arrhizus* PCR C_p**	***R. microsporus* PCR C_p**	***Mucor* spp. PCR C_p**	**HBG C_p**	
1	Fresh tissue	*Apophysomyces variabilis*	Not detected	Not detected	Not detected	37.08	Yes
2	FFPE tissue	*Apophysomyces variabilis*	Not detected	Not detected	Not detected	25.28	Yes
3	Fresh tissue	*Apophysomyces variabilis*	Not detected	Not detected	Not detected	30.21	Yes
4	FFPE tissue	*Cunninghamella bertholletiae*	Not detected	Not detected	Not detected	31.19	Yes
5	Fresh tissue	*Cunninghamella bertholletiae*	Not detected	Not detected	Not detected	36.46	Yes
6	Fluid	*Cunninghamella bertholletiae*	Not detected	Not detected	Not detected	28.82	Yes
7	Fresh	*Cunninghamella bertholletiae*	Not detected	Not detected	Not detected	32.90	Yes

8	Fluid	*Cunninghamella echinulata*	Not detected	Not detected	Not detected	26.32	Yes
			Not detected*	Not detected*	Not detected*	27.21*	Yes
9	Fluid	*Cunninghamella spp*	Not detected	Not detected	Not detected	22.83	Yes
10	FFPE tissue	*Lichtheimia corymbifera*	Not detected	Not detected	Not detected	37.55	Yes
11	Fluid	*Rhizomucor miehei*	Not detected	Not detected	Not detected	25.25	Yes
12	Fresh tissue	*Rhizomucor pusillus*	Not detected	Not detected	Not detected	35.23	Yes
13	Fresh tissue	*Rhizomucor pusillus*	Not detected	Not detected	Not detected	35.83	Yes
14	Fresh tissue	*Rhizomucor pusillus*	Not detected	Not detected	Not detected	23.94	Yes
15	BAL	*Rhizomucor pusillus*	Not detected*	Not detected*	Not detected*	28.7	Yes
16	FFPE tissue	*Rhizomucor pusillus*	Not detected	Not detected	Not detected	32.06	Yes
17	Fresh tissue	*Rhizomucor pusillus*	Not detected*	Not detected*	Not detected*	30.69	Yes
18	Fresh tissue	*Sakenaea vasiformis cx*	Not detected	Not detected	Not detected	31.63	Yes
19	Fresh tissue	*Sakenaea vasiformis cx*	Not detected	Not detected	Not detected	21.53	Yes
20	BW	*Syncephalastrum racemosum*	Not detected	Not detected	Not detected	31.38	Yes

BAL = bronchioalveolar lavage; BW = bronchial washing; C_p = crossing point; CSF = cerebrospinal fluid; FFPE = formalin fixed paraffin embedded; FNA = fine needle aspirate; HBG = human β globin; PCR = polymerase chain reaction; * indicates new DNA extraction performed on archived clinical specimens.

Appendix 4

Limit of Detection

Pan-Mucorales PCR

Organism	Dilution	Pan-Mucorales PCR C_p	HBG C_p
Rhizopus arrhizus	10^3	24.67	24.83
	10^2	29.60	25.36
	10^1	31.62	26.54
	10^0	Not detected	26.39
Rhizopus microsporus	10^3	24.98	25.55
	10^2	29.08	26.16
	10^1	35.23	26.21
	10^0	35.63	26.89
***Mucor* spp.**	10^3	24.23	24.89
	10^2	27.34	25.74
	10^1	33.63	26.40
	10^0	35.65	26.37
Lichtheimia corymbifera	10^3	26.31	25.94
	10^2	30.06	26.28
	10^1	32.45	26.30
	10^0	Not detected	26.23

C_p = crossing point; HBG = human β globin; PCR = polymerase chain reaction.

Multiplex genus/species-specific PCR

Organism	Dilution	***R. arrhizus*** **PCR C_p**	HBG C_p
Rhizopus arrhizus	10^3	26.57	25.72
	10^2	30.62	26.46
	10^1	32.09	26.63
	10^0	Not detected	26.51

C_p = crossing point; HBG = human β globin; PCR = polymerase chain reaction.

Organism	Dilution	***R. microsporus*** **PCR C_p**	HBG C_p
Rhizopus microsporus	10^3	29.66	26.32
	10^2	29.21	26.46
	10^1	33.25	26.50
	10^0	Not detected	27.05

C_p = crossing point; HBG = human β globin; PCR = polymerase chain reaction.

Organism	Dilution	***Mucor*** **spp. PCR C_p**	HBG C_p
***Mucor* spp.**	10^3	28.89	25.82
	10^2	31.94	26.19
	10^1	38.01	26.43
	10^0	Not detected	26.54

C_p = crossing point; HBG = human β globin; PCR = polymerase chain reaction
